# Supplementary material for: Nitrogen eutrophication particularly promotes turf algae in coral reefs of the central Red Sea
Source: PeerJ. 2020 Apr 2;8:e8737. doi: 10.7717/peerj.8737 (PMC7130110; doi:10.7717/peerj.8737)
Supplement: Supplemental Information 1 — Fertilizer: directly at the pin, Center: 25 cm towards the communities. *Indicates measurements from the reef water column. [file peerj-08-8737-s001.docx]

| **Location** | **Time reference** | **Water-Temperature [°C]** | **Distance from  fertilizer [cm]** | **NO_2_  [μM]** | **PO_4_  [μM]** | **NO_3_ [μM]** | **NH_4_  [μM]** | **DIN**  **[μM N]** | **DIN/PO_4_** |
| --- | --- | --- | --- | --- | --- | --- | --- | --- | --- |
| **Background*** | Start | 24.90 | --- | 0.04 | 0.06 | 0.34 | 0.13 | 0.51 | 8.37 |
| **Center** | 2 weeks |  | 25 | 0.02 | 0.06 | 0.91 | 0.20 | 1.14 | 22.37 |
| **Fertilizer** | 2 weeks |  | 0 | 0.03 | 0.44 | 5.68 | 1.34 | 7.06 | 17.00 |
| **Background** | 2 weeks | 26.27 | 200 | 0.01 | 0.09 | 0.27 | 0.05 | 0.33 | 3.53 |
| **Center** | 4 weeks |  | 25 | 0.06 | 0.12 | 1.07 | 0.24 | 1.37 | 11.68 |
| **Fertilizer** | 4 weeks |  | 0 | 0.06 | 0.57 | 5.52 | 1.66 | 7.24 | 13.99 |
| **Background** | 4 weeks | 26.48 | 200 | 0.07 | 0.15 | 0.33 | 0.09 | 0.49 | 3.36 |
| **Center** | 6.5 weeks |  | 25 | 0.03 | 0.20 | 1.16 | 0.21 | 1.41 | 9.22 |
| **Fertilizer** | 6.5 weeks |  | 0 | 0.05 | 0.30 | 5.04 | 1.49 | 6.59 | 40.79 |
| **Background** | 6.5 weeks | 27.34 | 200 | 0.03 | 0.06 | 0.09 | 0.09 | 0.20 | 3.21 |
| **Background*** | 8 weeks | 28.04 | --- | 0.05 | 0.07 | 0.36 | 0.16 | 0.56 | 13.47 |
