# Supplementary material for: Nitrogen eutrophication particularly promotes turf algae in coral reefs of the central Red Sea
Source: PeerJ. 2020 Apr 2;8:e8737. doi: 10.7717/peerj.8737 (PMC7130110; doi:10.7717/peerj.8737)
Supplement: Supplemental Information 2 — SD indicates the standard deviation (number of replicates: 4). [file peerj-08-8737-s002.docx]

|  | **δ^15^N** | **% N** | **δ^13^C** | **% C_org_** | **C_org_/N** |
| --- | --- | --- | --- | --- | --- |
| **Mean** | 16.326 | 7.722 | -27.027 | 6.166 | 0.800 |
| **SD** | 0.513 | 0.416 | 0.859 | 0.328 | 0.046 |
