# Supplementary figures and images for: Nitrogen eutrophication particularly promotes turf algae in coral reefs of the central Red Sea
Source: PeerJ. 2020 Apr 2;8:e8737. doi: 10.7717/peerj.8737 (PMC7130110; doi:10.7717/peerj.8737)

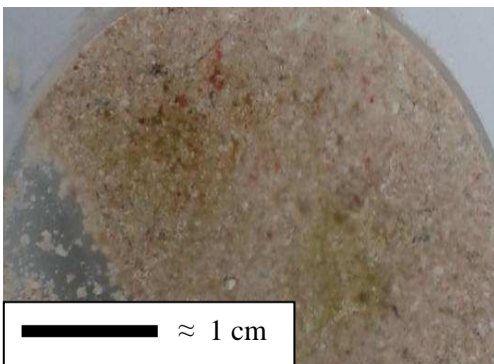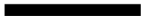

$\approx 1$  cm

Supplement: Supplemental Information 3 [file peerj-08-8737-s003.pdf]

$\delta^{13}\text{C}$

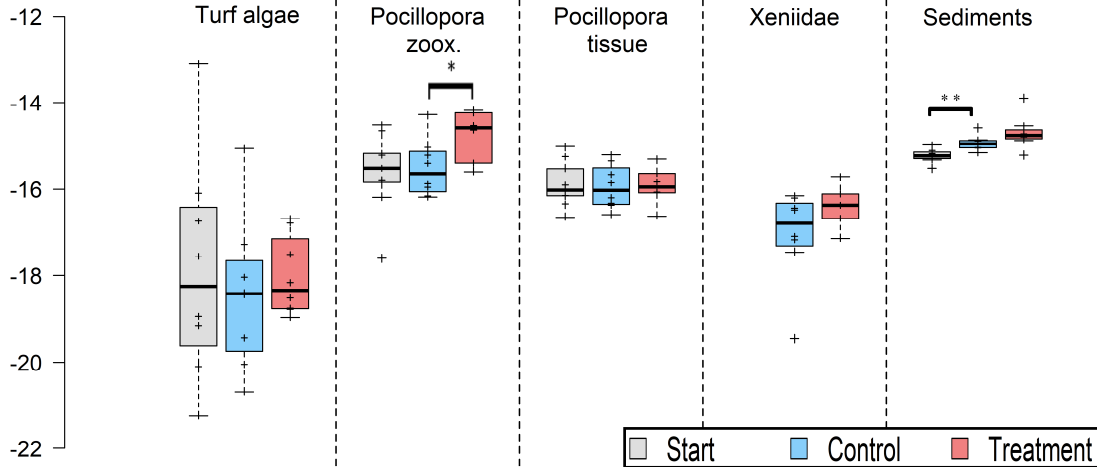

Supplement: Supplemental Information 4 — Asterisks indicate significant differences (*p < 0.05, **p < 0.005, ***p < 0.001). [file peerj-08-8737-s004.pdf]
